# Multiple scalp metastases from colonic neuroendocrine carcinoma: case report and literature review

**DOI:** 10.1186/1471-2407-14-305

**Published:** 2014-05-01

**Authors:** Shao-min Wang, Meng Ye, Shu-min Ni

**Affiliations:** 1 Department of Medical Oncology, Affiliated Hospital, School of Medicine, Ningbo University, No. 247 Renmin Road, Ningbo 315020, Zhejiang Province, China

**Keywords:** Colon cancer, Neuroendocrine carcinoma, Scalp, Metastasis

## Abstract

**Background:**

Colonic neuroendocrine neoplasms (NENs) are relatively rare tumors with an incidence rate of 0.11–0.21/100,000. NENs account for approximately 0.4% of colorectal neoplasms. Cutaneous metastases of colonic neuroendocrine carcinomas (NECs) are very infrequent, while cases of scalp metastasis are even fewer. Cutaneous metastases are more rare than visceral metastases and usually develop later; therefore, cutaneous metastases as initial distant metastases can be easily overlooked. This is the second case report of a colonic NEC with scalp metastasis. Compared with the previous case, in this instance scalp metastasis developed before visceral metastasis, and the cutaneous lesions were confined to the scalp alone.

**Case presentation:**

A 62-year-old Chinese man, who had undergone radical surgery for a “locoregional” colonic NEC one and half months before, came to our hospital for adjuvant chemotherapy. We found multiple scalp nodules during physical examination. Moreover, these nodules had occurred and had not been detected prior to the patient undergoing radical surgery. The scalp nodules proved to be metastases from colonic NEC as determined using pathological and immunohistochemical examinations following lumpectomy. After one and half months, visceral metastases were detected in this patient. Ultimately, the patient died two months later.

**Conclusions:**

In this report an unusual case of a colonic NEC with initial distant metastasis confined to the scalp is presented. This case is unusual because of the development of cutaneous metastasis before visceral metastasis. The scalp metastasis were initially overlooked, leading to inaccurate staging and radical surgery that was not curative. This demonstrates that distant metastasis can occur during the early phase of tumor growth in these aggressive lesions. Thus, the possibility of distant metastases should be assessed in the initial work up to avoid mistaken clinical staging especially when distant metastases occur only in skin.

## Background

Neuroendocrine neoplasms (NENs) are rare tumors originating from neuroendocrine cells and include a series of heterogeneous tumors. Their presentation can be indolent, low-grade malignant or high-grade malignant. These tumors can synthesize, store and secrete peptide hormones. Excessive hormones can cause corresponding clinical symptoms. According to the degree of differentiation, NENs have been divided into well differentiated NENs and poorly differentiated NENs in the 2010 World Health Organization classification of tumors of the digestive system, with the former being termed a neuroendocrine tumor (NET), and the latter, a neuroendocrine carcinoma (NEC) [1]. Cases of colonic NECs are very unusual and data from the Norwegian Registry of Cancer from 1993–2004 showed that the incidence rate of colonic NENs was 0.25/100,000 and 31.5% of colonic NENs were classified as NECs [2]. Distant metastasis of NECs mostly occurred in the viscera, while cases of scalp metastasis were rarely reported. A literature review in PubMed revealed only four case reports [3-6], however, primary colonic NEC was present in only one case [6]; these cases are summarized in Table 1.

**Table 1:** Cases of scalp metastases of NEC reported in the literature

**Ref**	**Sex/Age**	**Primary tumor**	**Metastasis to other sites**	**Cutaneous metastases before visceral metastases**	**Number of scalp metastasis**
[3]	F/31	Uterine cervix	Bone, lung, pleura, spinal cord, lymph nodes	No	2
[4]	F/33	Uterine	Cutaneous metastases of chest, back, abdomen, axilla and neck	Yes	>1
[5]	M/20	Bladder	Lung, retroperitoneal lymph nodes, skin of other sites	No	1
[6]	M/42	Colon	Liver, sphenoid, cavernous sinuses, bone, cutaneous metastasis of shoulder	No	1
Our case	M/62	Colon	Liver, pancreas, lymph nodes	Yes	>3

We now report a second case of a colonic NEC with multiple scalp metastases. Furthermore, this case has three features: (1) cutaneous metastases developed only in the scalp with no cutaneous metastases found at other sites; (2) there were multiple scalp metastases (>3); and (3) scalp metastases developed before visceral metastases. This case report, therefore, will focus attention on scalp metastases of an NEC. We have also reviewed the literature concerning gastroenteropancreatic neuroendocrine carcinomas (GEP-NECs) in this article.

## Case presentation

The patient was a 62-year-old man who was admitted to a local hospital because of abdominal pain and abdominal distension. Colonoscopy revealed a colon mass and a computed tomography (CT) scan of the abdomen showed that the mass infiltrated the serosa of the transverse colon, which was adjacent to the omentum majus with mesenteric lymph node metastases. The patient then went to a cancer hospital where a CT scan of his thorax and abdomen showed no distant metastases. Radical surgery was performed to remove his colon cancer on Feb 16th, 2013. Because the primary tumor was removed in a different hospital, we were unable to acquire a specimen of the resected primary tumor, however, we were able to obtain the pathological examination report. According to the report, the primary tumor was a poorly differentiated NEC of the right colon (grade 3) with a mass size of 9 × 5 × 3.5 cm. The tumor infiltrated the full thickness of the intestinal wall and extended into the extraserosal fibrous and adipose tissue, and 5/19 excised lymph nodes were positive for metastases. There were vessel cancer emboli, neural invasion, four cancer nodules around the tumor, four cancer nodules on the mesenterium and five cancer nodules on the omentum majus. The immunohistochemical report showed that tumor cells were stained positive for cytokeratin AE1/3, cytokeratin CAM5.2, chromogranin A (CgA), synaptophysin (Syn), CD56 antigen, CDX-2 and Ki-67 (approximately 30-50%).

**Figure 1:**
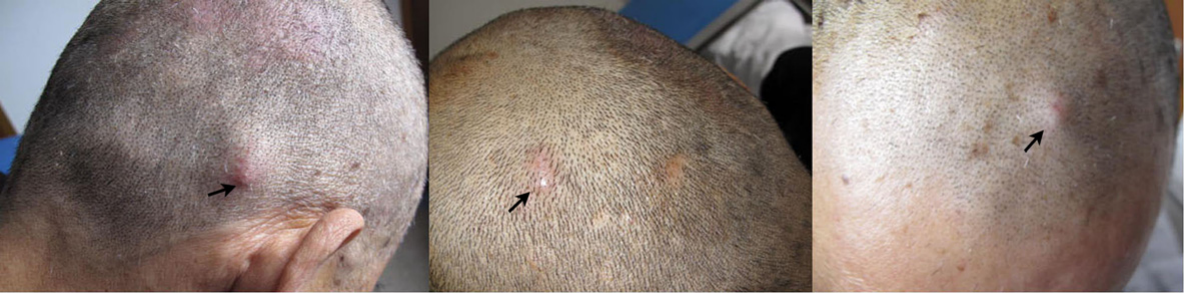
Multiple reddish nodules in the subcutis of the scalp.

**Figure 2:**
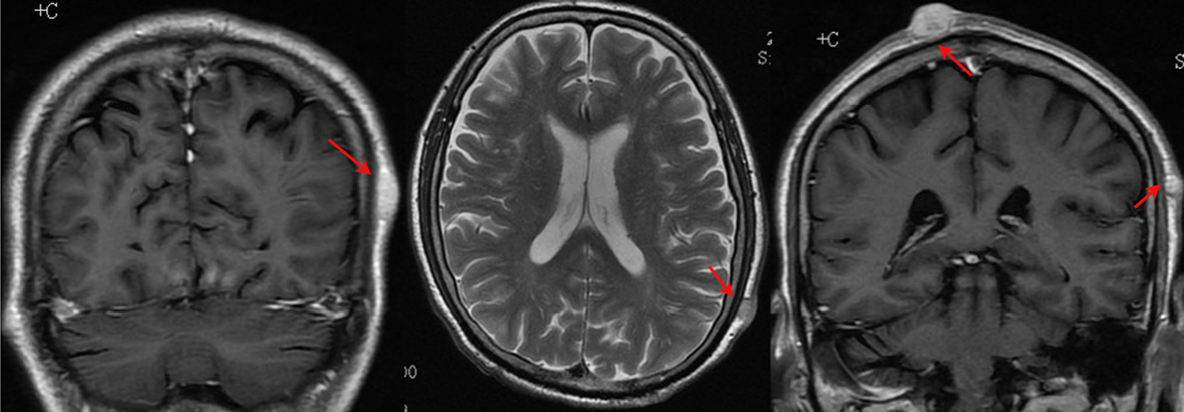
Head MRI showed multiple lesions in the soft tissue of the scalp.

The patient came to our hospital for adjuvant chemotherapy on March 28th, 2013. Multiple nodules in the scalp were found using physical examination (>3) (Figure 1) and no cutaneous nodules were palpated at other sites. The patient recalled that he had some reddish papules on his scalp before surgery, but he did not mention them to the surgeon. These papules became gradually larger and formed multiple nodules. Laboratory tests done on admission showed that carbohydrate antigen 19-9 was 51.27 U/mL (normal, <37 U/mL), carbohydrate antigen 125 was 95.09 U/mL (normal, <35 U/mL) and neuron-specific enolase (NSE) was 123.9 μg/L (normal, <17 μg/L). Magnetic resonance of the head showed multiple space-occupying lesions with a rich blood supply in the soft tissue of his scalp (Figure 2). Our patient received one cycle of systemic chemotherapy with a regimen of etoposide 100 mg/m^2^ IV on days 1-3 and cisplatin 25 mg/m^2^ IV on days 1-3.

**Figure 3:**
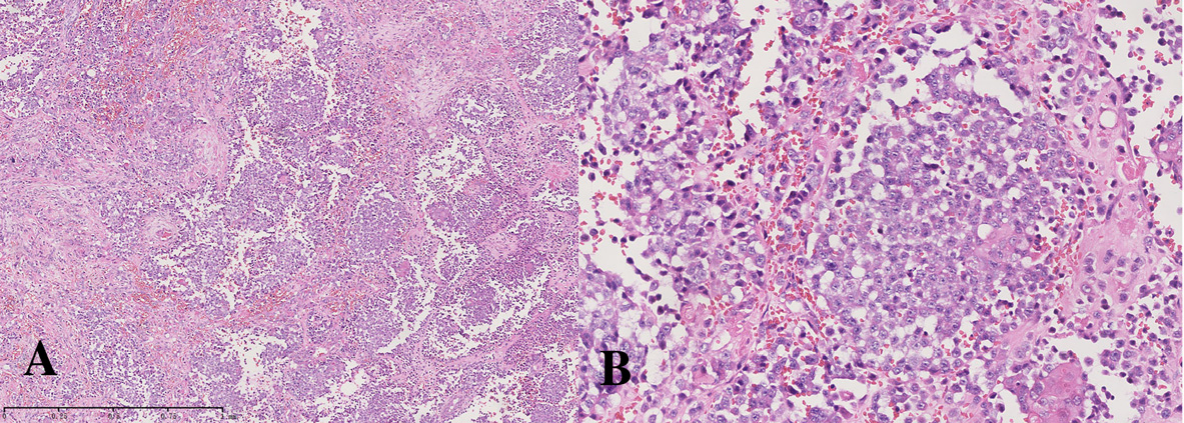
**Microscopic features of scalp nodule. (A)** Tumor cells arranged in diffuse and nesting patterns in the subcutis (hematoxylin-eosin staining, ×50). **(B)** Irregular and small to medium-sized tumor cells with scanty cytoplasm, hyperchromatic nuclei and distinct nucleoli in some cells (hematoxylin-eosin staining, ×400).

**Figure 4:**
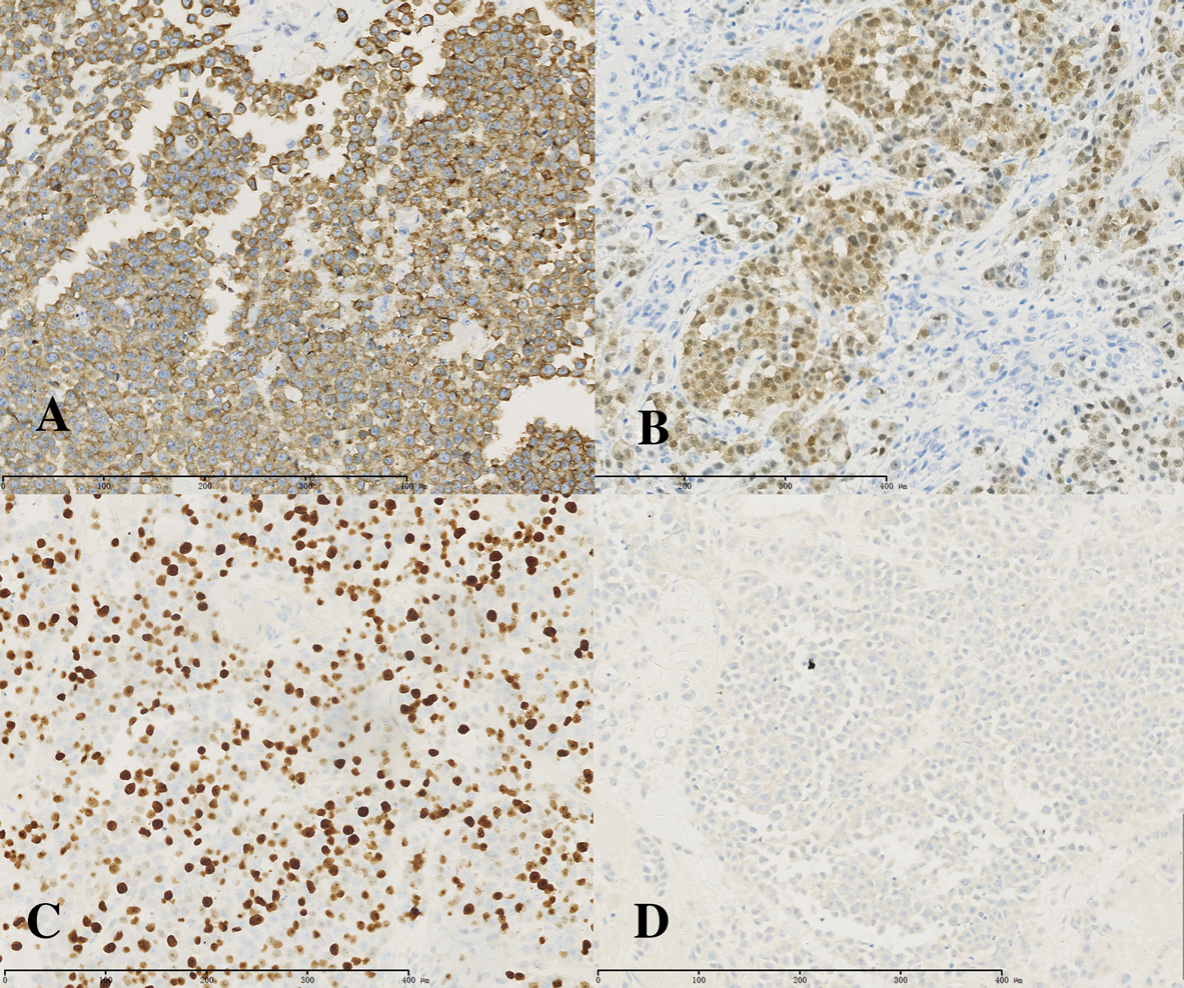
**Immunohistochemical analyses of tumor cells. (A)** Strongly positive staining of Syn (×200). **(B)** Strongly positive staining of CDX2 (×200). **(C)** Ki-67-positive nuclei (×200). **(D)** Weakly positive staining of CD56 (×200).

**Figure 5:**
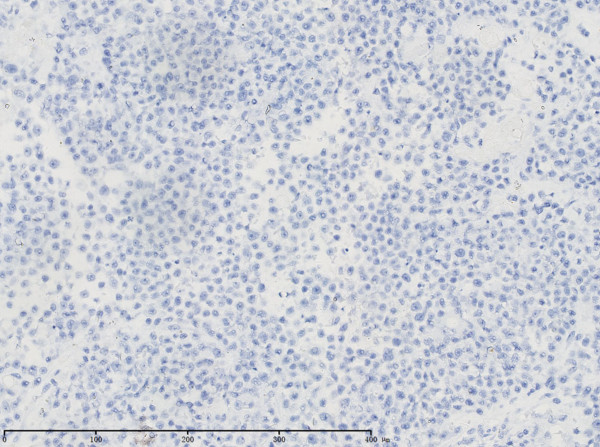
Immunohistochemical analysis showed that tumor cells were negative for CgA (×200).

He underwent a lumpectomy and transfer of a skin flap after chemotherapy. The largest nodule was about 2.5 cm in diameter, and it was removed during surgery. Pathological examination of the scalp nodule showed that the size of tumor was 2 × 2 × 1 cm. Hematoxylin-eosin staining showed that the scalp metastasis was composed of small to medium-sized cells with minimal cytoplasm, irregularly shaped nuclei with granular chromatin and prominent nucleoli in some cells. Tumor cells were arranged in diffuse and nesting patterns and mitoses were numerous (24 mitoses per 10 high power fields) (Figure 3). Immunohistochemical analyses showed that tumor cells stained positive for CD56 (weak), Syn (diffuse), CDX-2 (strong), pan-cytokeratin, cytokeratin 8, cytokeratin CAM5.2 and Ki-67, with the Ki-67 index reaching up to 60% (Figure 4). On the other hand, tumor cells stained negative for CK20, transcription termination factor 1 (TTF-1), melanoma antibody (HMB45) and CgA (Figure 5). On the basis of the pathological examination and medical history, the metastatic tumor of the scalp was considered to be a poorly differentiated carcinoma that originated from the colon with necrosis and neuroendocrine differentiation (NEC grade 3).

But other scalp nodules became larger and new nodules appeared after surgery. The patient received one additional cycle of the same chemotherapy regimen after April 23rd, 2013. Tumor progression was considered to have occurred once scalp nodules further increased in size and NSE levels further rose after chemotherapy. A CT scan of the patient’s abdomen was performed on May 14th, 2013. The scan showed that he had liver, pancreatic and lymph node metastases. Once visceral metastases were found, the patient received subcutaneous injection of octreotide acetate 0.1 mg per 8 hours for three days as of May 26th, 2013, and intramuscular injection of 20 mg octreotide acetate microspheres every 28 days three days later. Finally the patient received palliative and supportive treatment, and died of disease progression one month later.

## Conclusions

The gastrointestinal tract constitutes the most common site of origin of extra-pulmonary NECs, which can originate anywhere in the gastrointestinal tract [7]. The incidence rate of GEP-NENs is about 2–5/100,000 [8,9]. Functional NET is often accompanied by corresponding neuroendocrine symptoms. On the contrary, NECs are usually non-functional and without neuroendocrine symptoms. As a result, early-stage patients with NECs often have no specific signs or symptoms making early diagnosis very difficult, such that a majority of these patients already have disseminated disease at diagnosis [10-12]. The annual incidence of colonic NENs is 0.11–0.21/100,000 and NENs make up 0.4% of colorectal neoplasms [1]. Consequently when a mass is discovered in the colon, we seldom consider a diagnosis of a colonic NEC; rather a diagnosis of a colon adenocarcinoma is considered first. NSE has been shown to have a high diagnostic value in NECs [13,14]. The likelihood of an NEC is high when a patient has a colon mass that is accompanied by elevated serum NSE. At the time of initial diagnosis, extensive metastases usually do not appear in colonic adenocarcinoma, but distant metastasis often developed in NECs because of its invasive nature.

When this case was first diagnosed from the CT scan, this patient was considered to have regional disease and underwent radical surgery with curative intent. If scalp metastases had been found before surgery, a palliative operation may have been more suitable for this patient. Because of the high incidence of distant metastasis in NECs, when the patient was diagnosed with an NEC, a comprehensive preoperative physical examination and staging evaluation were necessary. When morphological imaging fails to find distant metastasis and distant metastasis is highly suspected, a positron-emission tomography-CT (PET-CT) examination should be done to acquire accurate clinical staging. Since cutaneous metastasis are infrequent in NECs and often overlooked, scalp metastases were not found prior to surgery that resulted in wrong preoperative staging in this case. From the medical history of this patient, scalp metastases occurred earlier than visceral metastases. So when the initial diagnosis of a GEP-NEC was made, we should have paid attention to whether scalp or cutaneous metastases were present.

In terms of the pathological diagnosis of NENs, grading of NENs is done according to the degree of proliferative activity of tumor cells, which is measured using two criteria, mitotic figures and (or) Ki-67 index. NENs of grade 1 or 2 are NETs, and NENs of grade 3 are NECs. Tumor cells of NETs are arranged in a characteristic organoid shape with nesting, and trabecular or gyriform patterns [15]. The cells are relatively uniform with round to oval nuclei having indistinct nucleoli and a coarsely granular chromatin pattern [1], expressing general markers of neuroendocrine differentiation (usually diffuse and intense CgA and Syn) [1]. In contrast, NECs have a more sheetlike or diffuse architecture with irregular nuclei and less cytoplasmic granularity [15]. Immunoexpression of neuroendocrine markers is more limited (diffuse expression of Syn, faint or focal staining for CgA) [1].

Representative NECs include small cell NECs (small cell carcinoma for short) and large cell NECs, while morphology resembles that of the corresponding lung tumor. Tumor cells of small cell carcinoma are small, round or oval like lymphocytes and some cells are elongated like fusiform; moreover cytoplasm is minimal. In this kind of tumor cell, nuclei are finely speckled and hyperchromatic, the nucleoli are inconspicuous and mitoses are numerous. Small cell carcinoma takes on a diffuse distribution or nesting arrangement, often accompanied by necrosis. Cells of small cell carcinoma are generally less than three times the size of lymphocytes. However, cells of large cell NECs are generally larger than three times the size of lymphocytes with coarse granular chromatin, prominent nucleoli and abundant cytoplasm. In large cell NECs, necrosis and mitoses are common, and the tumor takes on organoid, nesting, trabecular, rosette-like or palisading pattern.

Hematoxylin-eosin staining showed that in this case the scalp metastasis consisted of small to medium-sized tumor cells arranged in nesting patterns, which were in line with the features of an NEC. Further immunohistochemical analyses showed that tumor cells stained positive for Syn and CD56, which are neuroendocrine markers, while no staining for CgA indicated that the tumor was poorly differentiated. Nuclear staining of Ki-67 was positive and the Ki-67 index reached 60%, which was in accord with a grade 3 NEC. Cutaneous metastasis of NECs should be differentiated from Merkel cell carcinoma (MCC) and its origin should be investigated. CK20 is commonly positive in MCC, but it is usually negative in NECs at other sites [16]. CDX2 and TTF-1 are highly specific markers of gastrointestinal tract NENs and pulmonary NENs, respectively [17,18]. Tumor cells of scalp nodules were positive for CDX2, but negative for TTF-1 and CK20 in this case; therefore, scalp nodules were not MCC and originated from the colon.

Cases of GEP-NECs are rare and just a few large-scale clinical studies have been conducted. Because the clinical behavior of GEP-NECs is similar to that of small cell lung cancer (SCLC), treatment modalities are usually based on those prescribed for SCLC; therefore, adjuvant treatment with chemotherapy and/or radiotherapy should be given even after a definitive complete surgical resection [7]. The recommended regimen of adjuvant chemotherapy involves the combination of etoposide and cisplatin/carboplatin [19], and usually 4–6 cycles are administered [7]. Carboplatin and irinotecan seemed to be at least equally effective compared with cisplatin and etoposide [7,20,21]. Indeed, irinotecan was even better than etoposide in a multicenter retrospective analysis [22]. Second-line therapy for NECs is based on that used to treat relapsed SCLC, such as irinotecan, topotecan, paclitaxel, docetaxel, vinorelbine and gemcitabine [7]. In addition, a retrospective study showed that temozolomide, either alone or in combination with capecitabine or bevacizumab, attained a response rate of 33% as second-line therapy in patients with mostly GEP-NECs, but capecitabine or bevacizumab did not seem to have any additional effect compared to temozolomide [23]. Although the mammalian target of rapamycin signaling pathway inhibitor everolimus elicits a good response in patients with well differentiated NET, those with NECs are usually excluded from clinical trials (RADIANT 2, RADIANT3) because of their aggressive nature [24,25]. Thus, whether patients with NECs will respond to everolimus still needs to be confirmed. Somatostatin analogues (SSA) helped to control the syndrome and reduce circulating markers in GEP-NENs [26,27], but their antiproliferative effect was much less clear and disease stabilization was more common [28-30]. The combination of SSA and chemotherapy can be synergistic in the treatment of NETs [31], however, SSA with chemotherapy to treat NECs has only been evaluated in a phase II study [32]. Currently there is no evidence regarding the efficacy of the combination of SSA and chemotherapy in the treatment of GEP-NECs. Similarly, the vascular endothelial growth factor receptor-tyrosine kinase inhibitor sunitinib has not been evaluated in the treatment of NECs [33]. Our patient received etoposide and cisplatin as first-line chemotherapy, but did not respond. Since the patient had an NEC, SSA did not have any therapeutic effect.

Prognostic factors were also analyzed in NECs. Several studies have illustrated that the extent of the disease, patient performance status, Ki-67 index, maximal standard uptake value of a PET-CT examination and primary site were the best predictors of survival or response, however, patients with primary colonic NECs seemed to have shorter survival [8,11,12,34-36]. Blood levels of platelets and lactate dehydrogenase were strong predictors of survival as well [12]. The tumor of this patient, derived from the colon, was extensive with a high Ki-67 index, indicative of a poor prognosis. Postoperative survival of this patient was only five months. Primary drug resistance to chemotherapy occurred in this patient and receiving octreotide microspheres did not cause tumor remission. In conclusion, NECs are extremely high-grade malignancies, and therapeutic efficacy and prognosis are poor in patients with these performances.

## Consent

Written informed consent was obtained from the patient’s wife for publication of this case report and any accompanying images. A copy of the written consent is available for review by the Editor of this journal.

## Competing interests

The authors declare that they have no competing interests.

## Authors’ contributions

SMW reviewed the literature, prepared the data, and also drafted and revised the manuscript. MY made substantial contributions to the conception and design of the study, and also gave final approval of the version to be published. SMN participated in the design and coordination of the study. All authors read and approved the final manuscript.

## Pre-publication history

The pre-publication history for this paper can be accessed here:

http://www.biomedcentral.com/1471-2407/14/305/prepub
